# Autophagy Upregulation and Apoptosis Downregulation in DAHP and Triptolide Treated Cerebral Ischemia

**DOI:** 10.1155/2015/120198

**Published:** 2015-02-02

**Authors:** Yang Yang, Keqiang Gao, Zhiying Hu, Weiyun Li, Henry Davies, Shucai Ling, John A. Rudd, Marong Fang

**Affiliations:** ^1^Institute of Neuroscience, Zhejiang University School of Medicine, Hangzhou 310058, China; ^2^Department of Obstetrics and Gynecology, Hangzhou Red Cross Hospital, Hangzhou 310003, China; ^3^School of Biomedical Sciences, Faculty of Medicine, The Chinese University of Hong Kong, Shatin, New Territories, Hong Kong

## Abstract

It has previously been demonstrated that ischemic stroke activates autophagy pathways; however, the mechanism remains unclear. The aim of this study is to further investigate the role that autophagy plays in cerebral ischemia. 2, 4-diamino-6-hydroxy-pyrimidine (DAHP), for its nitric oxide synthase (NOS) inhibiting neuroprotective effect, and triptolide (TP), for its anti-inflammatory property, were selected to administer pre middle cerebral artery occlusion (MCAO). The drugs were administered 12 hours prior to MCAO. Both magnetic resonance imaging (MRI) and 2, 3, 5-triphenyltetrazolium chloride (TTC) staining showed that the drugs reduce the area of infarction. Immunoblotting analysis revealed increases in Beclin-1 and myeloid cell leukelia-1(Mcl-1) in treated rats. This could be a contributing factor to the reduction in autophagy induced damage. Immunochemistry and western blot showed that mTOR expression in treated rats was marginally different 24 h after injury, and this could also be significant in the mechanism. Furthermore, terminal deoxynucleotidyl transferase- (TdT-) mediated dUTP nick end labeling (TUNEL) staining proved that the drugs are effective in reducing apoptosis. The upregulation of Beclin-1 and Mcl-1 and downregulation of Bcl-2, caspase-3, and the Bcl-2/Beclin-1 ratio infer that the neuroprotective effect of DAHP and TP act via the mediation of autophagy and apoptosis pathways.

## 1. Introduction

Stroke remains the third leading cause of death in industrialized countries, with an incidence of approximately 0.25%–4% and a mortality rate of around 30% [1]. And it is becoming an evermore prominent global threat [2]. Ischemic stroke occurs when there is an acute blockage of arterial blood flow to the brain tissue. Thus, minimising infarction area and the generation of new neuronal cells in the injured brain are considered important strategic approaches [3]. Developing consistently effective therapy for ischemic stroke is a major challenge [4].

Brain edema is the first indication to the level of the ensuing disease course and the development of MRI and CT; brain edema has become one of the major determinate factors of livability in patients beyond the first few hours after stroke [1]. With regard to the pathological mechanisms of cerebral ischemia, it has been well established that caspase signaling, inflammatory factors, and excitotoxicity represent the main causes of neuronal apoptosis. Caspase inhibitors and antioxidants that are associated with the absence of complete biochemical and morphological characteristics of neuronal apoptosis have proved to be ineffective [5]. In light of this, we indicate that there must be other important cell death pathways that contribute to the pathophysiology [5, 6].

Recently, the possible role of autophagy in neurodegenerative diseases and tumor suppression has increasingly been examined. It is possible that diseases such as Huntington's, Parkinson's, and Alzheimer's could result due to autophagy deficiency. It has been reported that autophagy is not only activated in neurons by closed head injury or focal cerebral ischemia but also by hypoxic or excitotoxic stimuli [7, 8]. It is now a widely held view that autophagy can be viewed as a double-edged sword. To be more specific, it can be protective when activated by mild physiological stressors yet detrimental to neuronal survival when overactivated, leading to a series of fateful consequences [5].

It is known that autophagy can be rapidly upregulated in many processes such as ischemia [9], but the exact mechanisms underlying autophagy in cerebral ischemia remain unclear. It has been proved, however, that Nampt regulates autophagy in neurons upon cerebral ischemia through the TSC2 Ser1387-TOR-S6K1 signaling pathway via aSIRT1-dependent manner [5]. Moreover, previous study has shown that autophagy inhibitors can attenuate the secondary thalamic damage that follows focal cerebral infarction. Apoptosis is also involved in the process [10]. According to several studies, Beclin-1 is a major participant involved in ischemia-induced autophagy and Mcl-1, an antiapoptotic protein member of the Bcl-2 family, regulates the balance between autophagy and apoptosis [11, 12]. Also, it has been hypothesised that Mcl-1 and Beclin-1 may operate together in the same cells after ischemic reperfusion; results showed that both LC3 and Beclin-1 were evident in ischemic brains between 4 and 72 hours after MCAO. The coexpression of Mcl-1 with Beclin-1 might attenuate Beclin-1 dependent autophagy during ischemic stroke in rats [13]. Some studies demonstrated that, as one component of the class III phosphatidylinositol kinase (PI3K), Beclin-1 has been proved to initiate autophagy through interacting with the other components of PI3K pathway. However, there has also been research elucidating that autophagy induced by regulation of the TSC2-mTOR-S6K1 signaling pathway promotes neuronal survival during cerebral ischemia [5, 12].

DAHP, an inhibitor of GTP-cyclohydrolase I (GTP-CHI), has been proved to have anti-inflammatory properties and neuroprotective effects in many diseases, including cerebral ischemia. Previous study shows that DAHP treatment significantly reduced infarction volume [14]. However, its mechanism remains unknown. According to one investigation into the mechanism of DAHP, the pretreatment effects are related to inhibition of biopterin (BH(4)) and nitric oxide (NO) production [15]. To date, there has been no research concerning DAHP and autophagy.

With regard to the other drugs selected for this study, TP is one of the major active components of the traditional Chinese herb* Tripterygium wilfordii* Hook.f. and it has been reported to have potent anti-inflammatory and immunosuppressive properties. Furthermore, its effect on the pathogenesis of neurological disorders, such as Parkinson's disease, has been researched and found to play a protective role on dopaminergic neurons through its ability to limit lipopolysaccharide-induced inflammatory damage. The NF-*κ*B signaling pathway has been reported to be involved in the protective effects of TP. Therefore, TP treatment should be considered as a potential therapy for cerebral ischemia/reperfusion injury [16, 17]. However, the particular protective effects of TP in cerebral ischemia are still unclear.

Firstly, preliminary experiments were done on DAHP and TP treated MCAO model rats. We have found that the neuroprotective effect of DAHP was from inhibition of BH(4) and NO production. This process may be involved in the apoptosis pathway. Preexperimental results also showed that NF-*κ*B signaling pathways may be involved in the effect of TP pretreatment on ischemia. The results also reaffirmed that the drugs induced inflammatory and apoptotic responses. Correspondingly, we concluded that DAHP and TP were associated with cell apoptosis.

## 2. Material and Methods 

### 2.1. Animal Preparation

All the animals used in this study were pathogen-free male Sprague–Dawley rats weighing 250~280 g, provided by the Animal Center of Zhejiang University. All rats had access to food and water and kept in an air-conditioned room with a constant temperature of 24 ± 1°C. They were kept under a 12 hour light/dark cycle in separated clean cages. All feeding and breeding operations used in this study were compliant with the National Institutes of Health (NIH) Guide for the Care and Use of Laboratory Animals guidelines, which have also been approved by the Ethics Committee for Use of Experimental Animals in Zhejiang University.

### 2.2. Experimental Groups

A total number of 60 male Sprague-Dawley rats were randomly separated into 5 groups. Each group had 12 rats, 6 of them for extracting proteins and the other 6 for perfusion. The experimental groups were normal rats (control group), DMSO group (vehicle group), MCAO group, DAHP treatment group, and TP treatment group.

### 2.3. Drugs Administration

TP was purchased from Merck (Darmstadt, Germany), and DAHP was obtained from Sigma (Sigma, St. Louis, MO, USA). TP was dissolved in dimethyl sulfoxide (DMSO) and then injected intraperitoneally at a dose of 0.2 mg/kg [18]. The time point of administration is 12 hours before the MCAO operation. The rats in DMSO group were given an equivalent dose of DMSO under the same approach. DAHP was also dissolved in DMSO and injected intraperitoneally at a dose of 0.5 g/kg [14, 19]. The time point is also 12 hours before ischemia, because it has been reported to be the optimum time for neuroprotection in neurodegenerative diseases [14].

### 2.4. Middle Cerebral Artery Occlusion

Temporary focal cerebral ischemia was induced by occlusion of the right MCAO. In brief, after anesthetising with 10% chloral hydrate intraperitoneally, a nylon thread, which is 0.26 mm in diameter with a distal cylinder, was inserted into the lumen of the external carotid artery and advanced to the origin of the middle cerebral artery (MCA). A 4-0-monofilament nylon suture was prepared for making the knots [5, 8]. And three knots were made on arteries. After 90 minutes, the nylon thread was then removed to allow reperfusion. All the animals were allowed to recover from anesthesia and were sacrificed the following day for histological analysis and immunoblotting [8].

### 2.5. MRI Examination

A 3.0-Tesla (T) MRI animal scanner Magnetom Trio with TIM system (Siemens, Erlangen, Germany) was used to examine the rats' brains. The rats' heads were subjected to a custom device, of which the inner diameter is 30 mm, for signal excitation and detection. MRI parameters were set as follows: TE = 92 ms, TR = 3620 ms, FOV = 8 × 8 cm, *M* = 256 × 256, NA = 2, thickness = 2 mm, and gap = 0 mm. And then, after optimal adjustment of contrast by analyzing “mean density value” in Image-Pro Plus 5.0 software (Media Cybernetic, Bethesda, MD, USA), we obtained the images of hemisphere intensity.

### 2.6. TTC Staining

TTC staining was exploited for detecting infarction volume and was conducted as described previously. In breif, rats were deeply anesthetised with 10% chloral hydrate intraperitoneally and sacrificed by decapitation. The brain was rapidly dissected and sectioned into six coronal blocks in an adult rat brain matrix (Kent Scientific Corporation) with an approximate thickness of 2 mm. These sections were stained by incubating them in a solution of 2% TTC (Sigma, St. Louis, MO, USA) for 30 min at 37°C in the dark and then a solution of 4% paraformaldehyde. TTC was dissolved in physiological saline solution. The unstained areas were considered to be the infract areas, as presented in the figures. The total infarction volume for each slice was calculated by including all brain slices. The possible interference of brain edema to infarction volume was corrected by the following standard method, the noninfarcted area of the ipsilateral hemisphere/total noninfarcted area (from both the ipsilateral and contralateral hemispheres) [20].

### 2.7. Immunohistochemistry

The first procedure of immunohistochemistry is to get the tissue prepared. Briefly, the rats were deeply anesthetized with 10% chloral hydrate and then perfused transcardially with 500 mL of 0.9% saline to flush the vascular system, followed by 4% paraformaldehyde in 0.01 M phosphate-buffered saline (PBS, pH 7.4). The brains were removed and soaked in a 4% paraformaldehyde solution. After 24 hours, the brains were transferred to a 30% sucrose solution. Frozen sections, of which the thickness is about 18 *μ*m, were incubated with 5% normal blocking serum for 1 h at room temperature (RT) and then overnight at 4°C with primary antibody as follows: anti-beclin-1 (rabbit, monoclonal, 1 : 100; CST#3495), while the negative control sections were incubated with 0.01 M PBS instead of primary antibody. The next day, after being washed in 0.01 M PBS for 5 minutes three times, sections were incubated for 60 min with secondary antibody, which is goat anti-rabbit IgG included in the kit [7]. Then 0.01 M PBS for 5 minutes five times before DAB detecting (UltraVision HP IHC detection kit, TL-015-QHD). After hematoxylin staining, the sections were washed with ddH_2_O for 20 minutes and differentiated using 1% hydrochloric acid alcohol.

### 2.8. Immunofluorescent Labeling and TUNEL Staining

The frozen sections were rinsed in 0.01 M PBS for 5 minutes. After oven drying at 37°C, sections were blocked with 5% normal goat serum for 1 h at room temperature (RT). Thereafter, they were incubated with the caspase-3 (1 : 100, CST #9665) primary antibodies at 4°C overnight. Negative control sections were incubated with 0.01 M PBS rather than primary antibody [13]. The next day, after being washed in 0.01 M PBS for 5 minutes three times, sections were incubated at RT for 1 h with FITC goat anti-rabbit IgG (1 : 100, Boster BA1105). After being washed three times, the sections were prepared for fluorescence. TUNEL staining was done on sections of each group. All the procedures are following the manufacturer's suggestions of the in situ cell death detection kit (Calbiochem, QIA39). Fluorescence signals were detected by a fluorescence microscope (Olympus BX51, NIKON, Japan) at excitation/emission wavelengths of 550/570 nm (Cy3, red) and 492/520 nm (FITC, Green and blue) [10].

### 2.9. Western Blot Analysis

Total protein was extracted with western blot lysis buffer containing 20 mM TRIS-HCl (pH 7.4), 1.5 M NaCl, 1%TritonX-100, 0.1%SDS, 1 mM Na_2_EDTA, and 1% PMSF (Phenylmethanesulfonyl fluoride, Beyotime ST505). The homogenate was then centrifuged at 12000 rpm for 20 min at 4°C and the supernatant was preserved at −80°C [10]. Protein concentration was determined using a BCA kit (KeyGEN, Nanjing, China). Twenty mg of protein from each sample was subjected to electrophoresis on 10% SDS-PAGE gel using a constant voltage [21]. Electrophoresis was performed at 100 V for 20 min, then 180 V for 40 min, and separated proteins were electrophoretically transferred to a polyvinylidene difluoride (PVDF) membrane in a Bio-Rad TransBlot apparatus, which was performed at 100V for 120 min. The blots were blocked with 5% rehydrated nonfat dry milk for 2 h at room temperature and then incubated with primary antibody overnight at 4°C. The primary antibodies were rabbit polyclonal antibody against *β*-actin (1 : 2000, Abmart #P30002), Beclin-1 (1 : 1000, CST#3495), mTOR (1 : 1000, EPITOMICS, p42345), and Mcl-1 (1 : 100, SANTA CRUZ, sc-819). The following day, after 5 minutes of TBST washing over 3 times, the blots were incubated with goat anti-rabbit IgG antibody (AURAGENE, SA009) for 2 hours at room temperature. The detection reagents were placed on the blot for 1 min and then exposed to Hyperfilm-ECL for 2–30 min and then exposed with Kodak X-OMAT films [8].

### 2.10. Statistical Analysis

The statistical differences between two groups were analyzed using the established* t*-test. One-way analysis of variance (ANOVA) was used to determine statistical significance with SPSS 16.0 program. The null hypothesis was rejected at the 0.05 level. GraghPad prism 5 was used to perform the histograms.

## 3. Results 

### 3.1. MRI Outcome Demonstrates That Both DAHP and TP Attenuate Cerebral Ischemia

**Figure 1:**
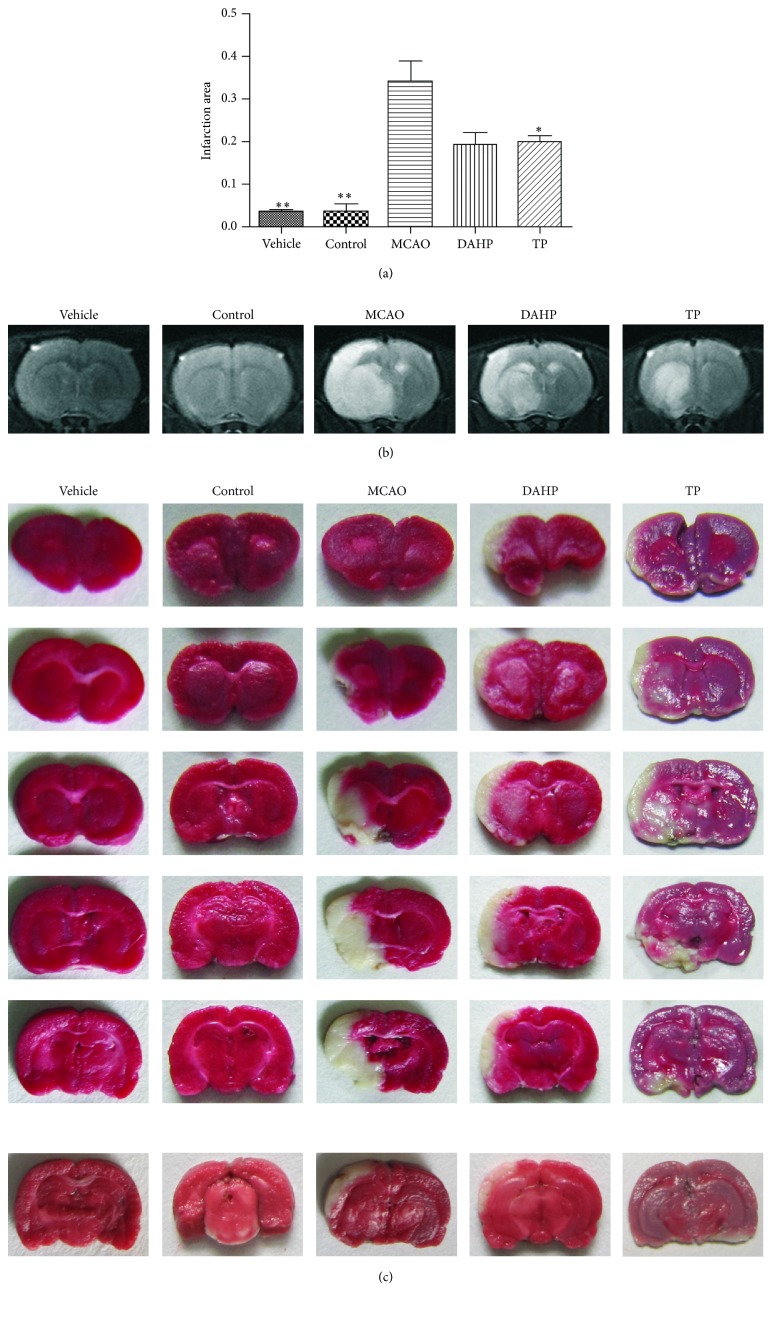
The representative images of TTC staining and MRI outcome show the effects of DAHP and TP on MCAO-induced focal cerebral ischemia in rats. (a) Mean change in infarction volume is significant in MCAO group (MCAO), DAHP treatment group (DAHP), and TP treatment group (TP) in comparison with the normal control (control). Infarction area was less in the treatment groups than the MCAO group, thus presenting their neuroprotective effects. Data is expressed as means ± SEM (*n* = 5–10); ^*^ *P* < 0.05, ^**^ *P* < 0.01, and ^***^ *P* < 0.001 compared with normal control. (b) Compared with vehicle group (vehicle) and control, the images show hyperintensity in MCAO, DAHP, and TP, among which MCAO has the largest hyperintensity areas. (c) The images of TTC staining in each group show the same tendency of ischemia infarction as the MRI results; DAHP and TP attenuate cerebral ischemia. TTC staining results revealed that the differences in infarction areas were significant in rats pretreated by DAHP and TP, which is consistent with MRI results.

MRI is considered the most promising and noninvasive approach for examining brain edema formation. With T2-weighted imaging, we can determine the edema induced by I/R to evaluate cerebral edema. As we can ascertain from the images in Figure 1(b), the MCAO group has obvious hyperintensity, while no hyperintensity was shown in the control group. Both the DAHP treatment group and TP treatment group have less hyperintensity than the MCAO group. From this result, we can deduce that both DAHP and TP attenuate cerebral ischemia [22].

### 3.2. TTC Staining Shows That DAHP and TP Treatments Can Reduce Infarction Volume Following Focal Brain Ischemia

Representative samples of TTC-stained brain sections are shown in Figure 1(c), and corresponding infarction volumes are shown in Figure 1(a). No cerebral infarction was observed in the sham group or the control group. A significant increase in infarction volume in the MCAO group can be seen. When compared with the DMSO group, there was a clear reduction in infarction volume in the DAHP treatment group and the TP treatment group (Figure 1(a)).

### 3.3. Immunofluorescent Labeling and TUNEL Staining Results Present a Decrease in Apoptosis after DAHP and TP Treatments

**Figure 2:**
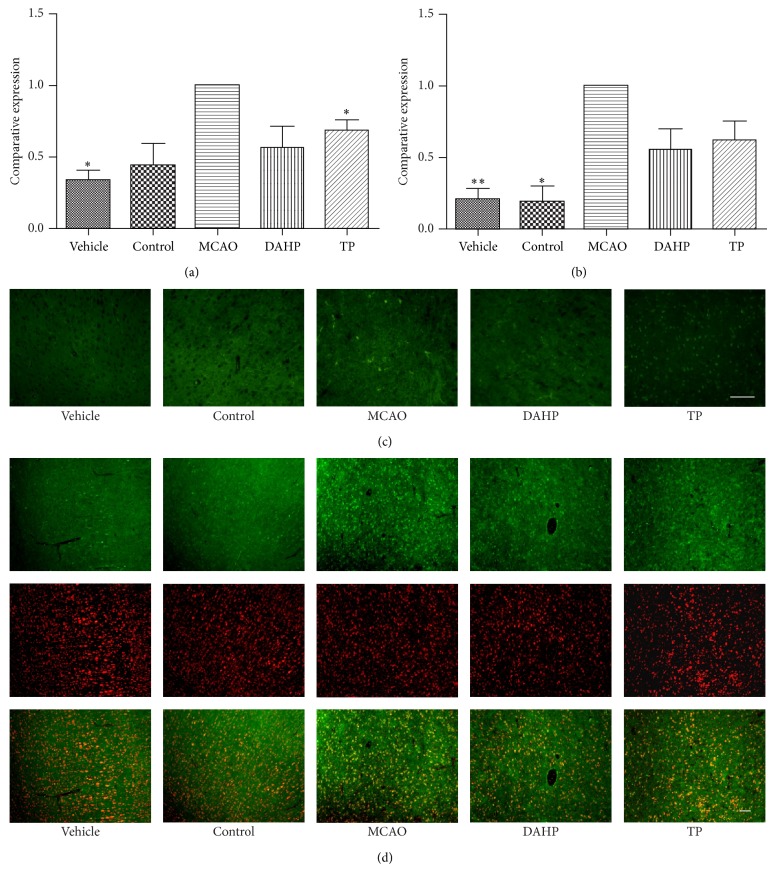
Apoptosis level detected by immunofluorescent labeling and TUNEL staining in each group. It can be seen from the images that both the expression of caspase-3 and TUNEL and, therefore, apoptosis, decrease in DAHP and TP. (a) The histograms show positive cells detected by TUNEL staining in each group. Values are means ± SEMs. ^*^ *P* < 0.05; ^**^ *P* < 0.001; ^***^ *P* < 0.0001; n.s.: no significance. (b) The histograms showed expression of caspase-3 in each group. Values are the means ± SEMs. ^*^ *P* < 0.05; ^**^ *P* < 0.001; ^***^ *P* < 0.0001; n.s.: no significance. (c) Compared with vehicle and control, the image shows more positive cells presented in MCAO, DAHP, and TP. Also MCAO owns the largest number of positive cells. (d) The images of expression levels of caspase-3 show similar change trends among each group in which both DAHP and TP decrease apoptosis. Bar = 100 *μ*m.

TUNEL-positive cells represent apoptotic cells. From Figure 2(c), we can see that the proportion of positive cells in MCAO group is markedly higher than the control group. The proportion of positive cells in the DAHP treatment group and the TP treatment group is significantly less than the MCAO group but still more than the control group. In addition, immunofluorescent labeling of caspase-3 showed a similar decrease in apoptosis in the DAHP treatment group and the TP treatment group. This can be seen in Figure 2(d). Caspase-3 expression in the DAHP treatment group and the TP treatment group was higher than the control group.

### 3.4. Immunochemistry and Western Blot Staining Reveal an Upregulation of Beclin-1 in DAHP and TP Treatments Groups

**Figure 3:**
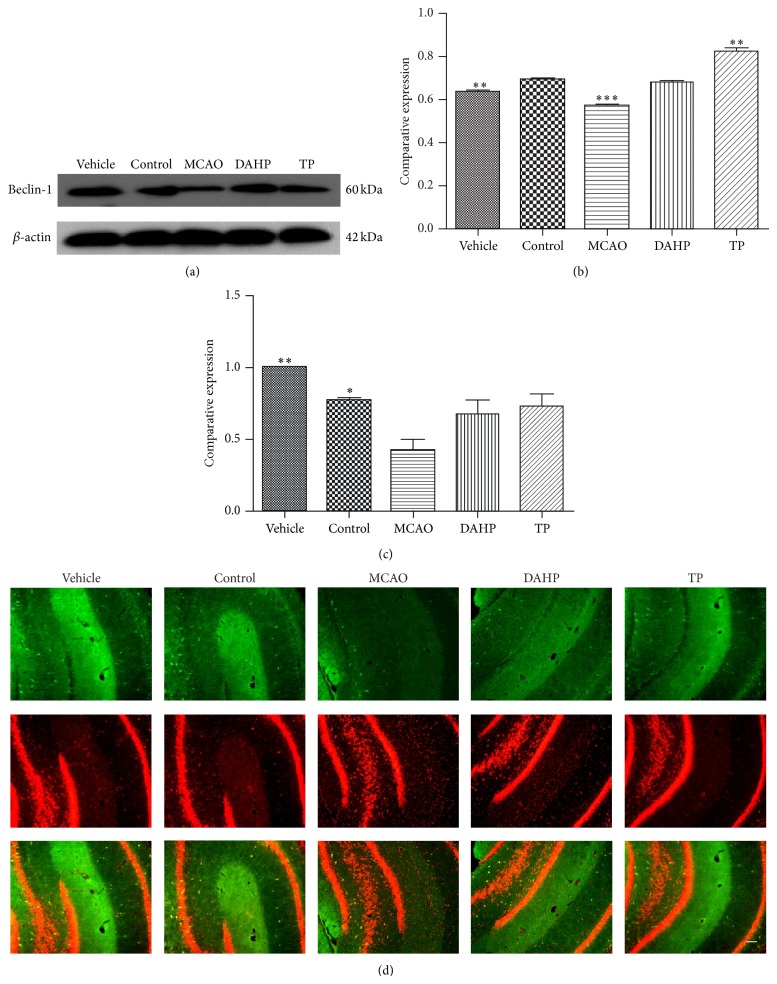
The expression level of Beclin-1 was detected by immunochemistry and western blot. Both immunochemistry and western blot staining present an increasing tendency in Beclin-1 expression in DAHP and TP and a decrease in MCAO. (a) There is no obvious difference between vehicle and control. The image shows a decrease in MCAO, while DAHP and TP were more or less back to the same level as vehicle and control. (b) There has been significance among mean changes in Beclin-1, as compared with vehicle and normal control; MCAO has the trend to descend. (c) The mean change in Beclin-1 expression detected by immunochemistry has significance, also owning comparison among MCAO, DAHP, and TP. (d) The immunochemistry staining shows similar changes in Beclin-1 expression in each group as in (a). Bar = 100 *μ*m.

Representative figures of the immunofluorescent and western blot staining results are shown in Figures 3(d) and 3(a), respectively. Previous research has proved that autophagy inhibition by Beclin-1 knockdown can attenuate secondary thalamic damage after focal cerebral infarction. In comparison with the MCAO group, a clearly elevated level of Beclin-1 was detected in the DAHP treatment group and the TP treatment group. Furthermore, both the western blot and the immunochemistry analysis showed that Beclin-1 expression was higher in the TP treatment group than the DAHP treatment group; results can be seen in Figures 3(b) and 3(c).

### 3.5. Mcl-1 Expression Showed the Same Change Tendency as Beclin-1 according to Western Blot Detection

**Figure 4:**
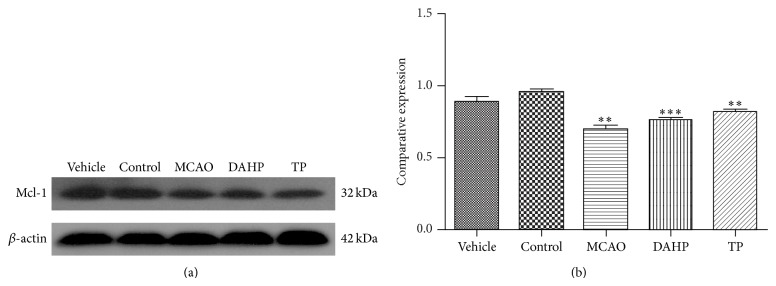
Western blot was conducted to detect the expression level of Mcl-1 among each group, which is consistent with Beclin-1 expression. Western blot staining presents an increasing tendency in Mcl-1 in DAHP and TP but a decrease in MCAO. (a) Compared with vehicle and control, the image shows a slight decreasing in MCAO, and the DAHP and TP were very likely to be the same level. (b) The mean change in Mcl-1 was significant. MCAO has a decrease, while DAHP and TP attenuated the tendency. DAHP and TP can be compared with normal control with a small fluctuation of 5%–10%. Values are the means ± SEMs. ^*^ *P* < 0.05; ^**^ *P* < 0.001; ^***^ *P* < 0.0001; n.s.: no significance.

From Figure 4(a), it can be seen that, much like Beclin-1 expression, Mcl-1 expression was lower in the MCAO group but elevated in DAHP and TP treatment groups. Therefore, in agreement with previous studies [10, 12], our western blot results also showed a similar change trend in Beclin-1, which can be seen in Figure 4(b). The expression of Mcl-1 in the MCAO group is lowest, then the two treatments groups and finally the control group. The similarity in the changes between Mcl-1 and Beclin-1 levels in each group indicates that the two proteins could be concomitantly involved in the regulation autophagy.

### 3.6. Western Blot and Immunochemistry Staining Reveal an Upregulation of mTOR after MCAO

**Figure 5:**
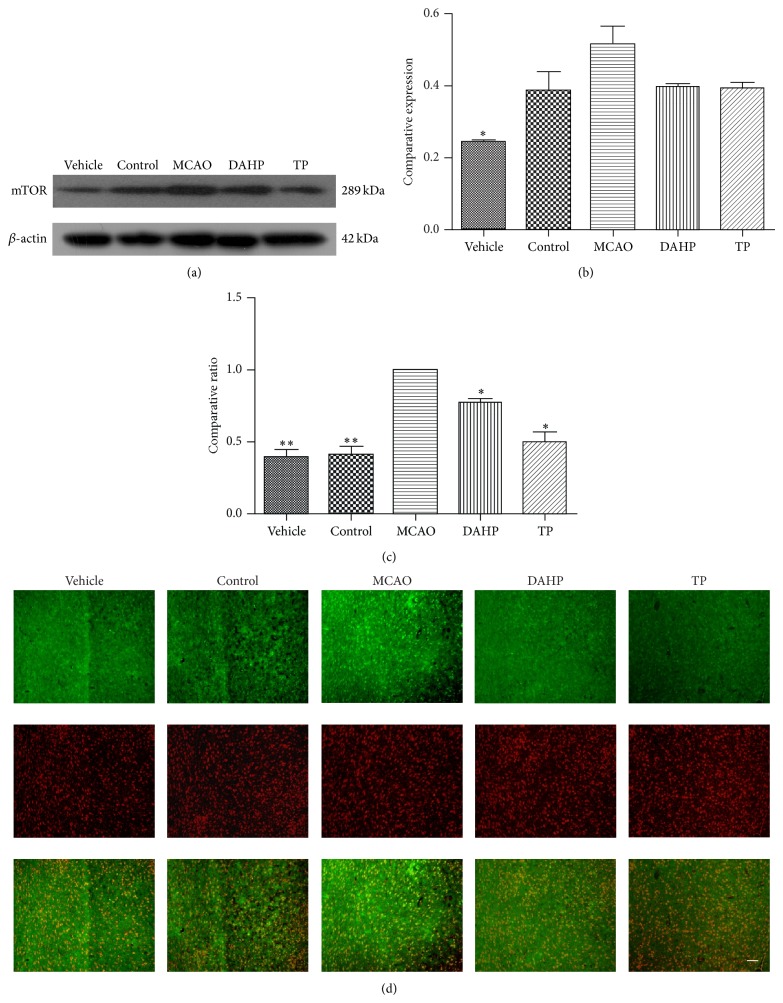
mTOR upregulation after MCAO was revealed under western blot and immunofluorescent staining. Western blot analysis of protein levels of mTOR and *β*-actin in the rat cortex derived from each group. (a) The images show that MCAO has an increase on vehicle, whereas DAHP and TP treatment groups are decreased. (b) Optical densities of respective protein bands were analyzed and normalized to the control (*β*-actin). Results were expressed as means ± SDs from three independent experiments. (c) From the histograms, the expression of mTOR in each group has a comparatively large fluctuation. Values are the means ± SEMs. ^*^ *P* < 0.05; ^**^ *P* < 0.001; ^***^ *P* < 0.0001; n.s.: no significance. (d) The immunofluorescent staining results were consistent with those of protein level differences, mentioned above in (a). MCAO has an increasing trend. Bar = 100 *μ*m.

As shown in Figures 5(a) and 5(b), western blot analysis revealed that mTOR expression levels were higher in the MCAO group than the vehicle and control groups. The DAHP treatment group and the TP treatment group were lower than the MCAO group but higher than the vehicle and control groups. The immunochemistry results presented the same phenomenon. From Figure 5(d), it can be seen that the positive staining area is greater in the MCAO group than the control group, while the DAHP treatment group and TP treatment groups were in between the two; corresponding changes can be seen in Figure 5(c). Both western blot and immunochemistry staining showed that mTOR was upregulated after MCAO and attenuated with DAHP treatment and TP treatment.

### 3.7. Immunofluorescent Analysis of Bcl-2 Expression Shows That DAHP and TP Have Antiapoptotic Effects

**Figure 6:**
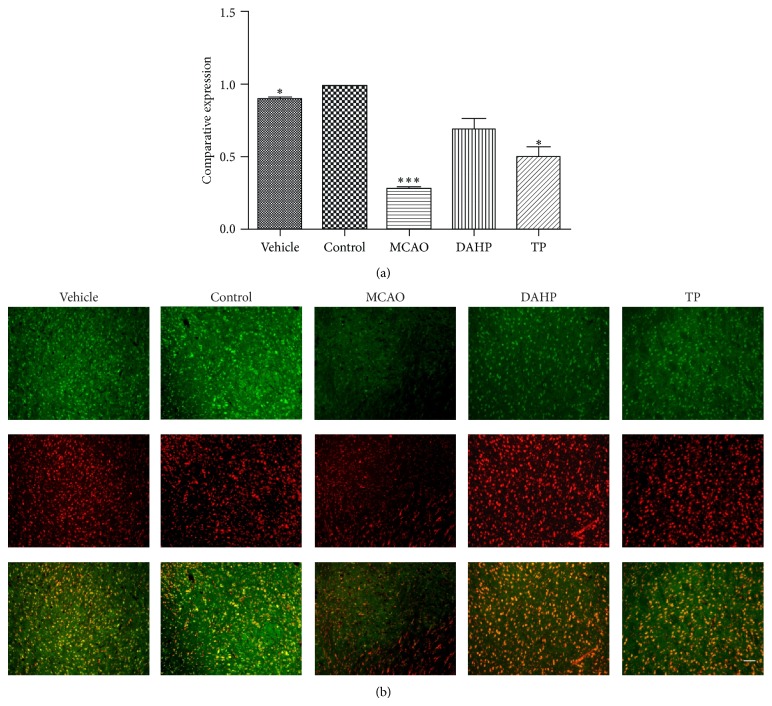
Immunofluorescent analysis of Bcl-2 expression represents antiapoptosis level changes. The expression levels of Bcl-2 were markedly different in each group. (a) In comparison with control, MCAO has only 30% of its Bcl-2 expression, DAHP has 75%, and TP has 60%. Values were presented with the means ± SEMs. ^*^ *P* < 0.05; ^**^ *P* < 0.001; ^***^ *P* < 0.0001; n.s.: no significance. (b) The immunofluorescent results showed a dramatic decrease in Bcl-2 expression in the MCAO group in comparison with the vehicle and control groups, while the DAHP group and TP group attenuated this change. Bar = 100 *μ*m.

The antiapoptotic protein Bcl-2 has been proved to play a role in Beclin-1 dependent autophagy. From Figures 6(b) and 6(a), it can been seen that the immunofluorescent results showed a dramatic decrease in Bcl-2 expression in the MCAO group in comparison with the vehicle and control groups, while the DAHP group and TP group attenuated this change. Our result is consistent with previous study, which demonstrated that Bcl-2 can inhibit Beclin-1 induced autophagy.

## 4. Discussion 

This present study used the MCAO model and two intraperitoneally administered drugs, DAHP and TP, selected due to their proven neuroprotective ability in certain diseases. MRI and TTC staining were exploited, and the result demonstrated that DAHP and TP can reduce the infarction area. Immunoblotting analysis shows that both the DAHP treatment group and the TP treatment group increased Beclin-1 and Mcl-1 levels after MCAO. Furthermore, TUNEL staining proved the drugs effective in reducing apoptosis. Thus, we concluded that DAHP and TP can regulate autophagy and apoptosis in MCAO model rats.

DAHP has been firmly proved to be play a role in inhibiting BH(4) and NO production. TP has been shown to have anti-inflammatory properties. Therefore, both DAHP and TP have neuroprotective potential in neurodegenerative diseases, including cerebral ischemia. Although much research has been done to explore the exact mechanism of ischemic stroke, it remains unclear whether a pretreatment of DAHP or TP could be considerably protective for cerebral ischemia. And the mechanism closely related with autophagy and apoptosis needs more investigation [21, 23]. Our present study employed a sophisticated MCAO model, aiming to testify the protective mechanism of DAHP and TP. Meanwhile, we are exploring the connections between protection and autophagy.

**Figure 7:**
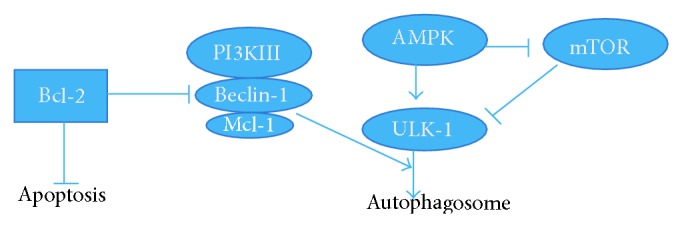
The Beclin-1 network regulates autophagy and apoptosis. DAHP and TP can attenuate cerebral ischemia through Beclin-1/Mcl-1 regulated autophagy pathways, which interacts with the apoptosis regulator Bcl-2.

The complete role that autophagy plays in the pathogeneses of some neurodiseases has yet to be agreed upon. However, researcher have uncovered that tumor necrosis factor-*α* (TNF-*α*) upregulates the expression of LC-3 and Beclin-1 induces autophagy by activating the JNK pathway and inhibiting the AKT pathway [24, 25]. Recent studies have found that some autophagy related proteins are directly involved in immune and inflammatory responses, throughout the whole autophagy process. We also know that macrophages contain Toll like receptor (TLR) ligand coated particles of phagosome and lysosome, and the fusion process for autophagy requires the participation of Beclin-1 and LC-3. Also, in dendritic cells, autophagy related proteins are involved in the fusion of lysosomal and TLR phagosomes, containing apoptotic cell antigen [26]. Beclin-1-PI3K compound, LC3, and Atg12 can be recruited to the gram negative bacteria vesicles [27]. It was from this that we concluded our hypothesis that each of the aforementioned factors may be concomitantly involved in autophagy's role in neuron destruction in cerebral ischemia (Figure 7).

To date, a consensus has not been reached on whether autophagy has protective or detrimental role in neuronal damage. Our results indicate that both apoptosis and autophagy play protective roles at the beginning [28, 29]. However, they become detrimental later on, after crossing the warning fence. Researches have shown that Beclin-1 interacts with Bcl-2 to inhibit autophagy, leading to a downregulation of apoptosis in the central nervous system (CNS). However, since Beclin-1 and Bcl-2 are separated, autophagy is always happening [30, 31]. Beclin-1 is important in autophagy related processes. The expression of Beclin-1 promotes autophagy. Thus, downregulated Beclin-1 expression reduces autophagic activity [9]. Beclin-1 plays an important role in all autophagy related procedures, except for the formation of autophagic vacuoles. Therefore, we suspect that Beclin-1 is a crucial protein regulating autophagy, but not necessary for autophagy to take place. Recent studies show that Beclin-1 works in both the autophagy and apoptosis processes. Bcl-2, an antiapoptosis factor, also has regulatory effects that interact with Beclin-1 to participate in the regulation of autophagy and apoptosis. From the above-mentioned statement about these two crucial factors, the interrelation of autophagy and apoptosis can be closely related with Beclin-1 and Bcl-2 interactions [31, 32]. We found a dramatic elevation in Beclin-1 expression levels in both the DAHP treatment group and the TP treatment group challenged by cerebral ischemia. This result is in agreement with previous literature data [21, 33].

Our results showed that the change in Mcl-1 expression followed the same trend as Beclin-1. And this phenomenon is in accordance with previous study, which demonstrated that Mcl-1 potentially modulated Beclin-1 induced autophagy. Mcl-1 is a key regulator of apoptosis during central nervous system development [21, 34]. Emerging data suggest that Mcl-1 is critical for the survival of different cells. It is an antiapoptosis gene in the Bcl-2 family, so inhibition of Mcl-1 promotes cell death [6, 35]. Many chemotherapeutic drugs induce apoptosis through downregulation of Mcl-1 expression in tumor cells to reach their therapeutic effects. And there has been an increasing amount of research on interdisciplinary studies between cancer and neurodegenerative diseases [36].

Our results demonstrate that apoptosis was clearly increased after MCAO, while autophagy was downregulated. Moreover, in the DAHP treatment group and the TP treatment group, neuroprotective roles were conducted through the downregulation of apoptosis and upregulation of autophagy, resulting in an attenuation of cerebral ischemia. In addition, there already existed papers on the effective treatments of these two drugs on other diseases. It has been well accepted that autophagy is a double-edged sword in ischemia [27], in that it can be moderately activated to promote cell survival under certain circumstances, like starving and hypoxia [29], yet it is disastrous when it is overactivated, leading to cell lysis and cell death. Therefore, we hypothesised that DAHP and TP achieve their protective effects in ischemic stroke by moderately regulating autophagy rather than disastrously overactivating it. Similarly, apoptosis was also appropriately adjusted, thus moderately regulating both apoptosis and autophagy. Therefore, only when autophagy is activated to within a certain range can the drugs be effective in treating cerebral ischemia.

From our experiment, we have proven our hypothesis on both a protein and a histological level. However, there were certain limitations on our experiments; for instance, the results were only from 24 h reperfusion. However, some other researches conducted parallel experiments and explored the relationship between reperfusion time points and treatment effects of certain drugs. Furthermore, there have already been studies indicating that autophagy exhibited the strongest effect at 72 h after reperfusion. Accordingly, we plan to conduct experiments with a larger timescale, the 72 h mark in particular.

## Abbreviations

MCAO: Middle cerebral artery occlusion
DAHP: 2, 4-Diamino-6-hydroxy-pyrimidine
Mcl1: Myeloid cell leukemia-1
MRI: Magnetic resonance imaging
TTC: 2, 3, 7-Triphenyltetrazolium chloride
TUNEL: Terminal dexynucleotidyl transferase- (TdT-) mediated dUTP nick end labeling
PI3K: Class III phosphatidylinositol kinase
GTP-CHI: GTP-cyclohydrolase I
DMSO: Dimethyl sulfoxide
PVDF: Polyvinylidene difluoride
TNF-*α*: Tumor necrosis factor-*α*
TLR: Toll like receptor
mTOR: Mammalian target of rapamycin.
